# Acute Intoxications Admitted to the Intensive Care Unit: A Retrospective Cohort Study

**DOI:** 10.1155/jt/8823675

**Published:** 2025-08-06

**Authors:** Martina Lombardo, Roberta Garberi, Emanuele Rezoagli, Matteo Pozzi, Francesco Bartoli, Roberto Rona, Giuseppe Citerio, Giuseppe Foti, Marco Giani

**Affiliations:** ^1^Department of Medicine and Surgery, University of Milano-Bicocca, Monza, Lombardy, Italy; ^2^Department of Emergency and Intensive Care, Fondazione IRCCS San Gerardo dei Tintori, Monza, Lombardy, Italy; ^3^Department of Neuroscience, Fondazione IRCCS San Gerardo dei Tintori, Monza, Lombardy, Italy

**Keywords:** acute intoxication, early recognition, ICU, multisubstance, single-substance

## Abstract

**Background:** Acute intoxications are a critical yet underexplored area in intensive care. Poisoning ranks among the leading causes of injury-related death, but limited data and the lack of clinical guidelines hinder prompt recognition and effective management. This study aims to describe intensive care unit (ICU) admissions for acute intoxications at an Italian tertiary hospital and to identify key factors associated with patient outcomes.

**Methods:** We conducted a retrospective cohort study of patients admitted for confirmed acute intoxication to the ICUs (general, cardiac, and neuro) at Fondazione IRCCS San Gerardo dei Tintori Hospital, Monza, from January 2009 to May 2024. Data included demographics, substance type, intoxication severity, treatments, and outcomes.

**Results:** Among 117 patients (126 intoxication episodes), intentional self-poisoning, often involving psychotropic drugs, was most prevalent. Multiple-substance intoxications made up 55.6% of cases, typically involving medications and ethanol, and were associated with shorter ICU stays and lower mortality than single-substance cases, where toxic agents like cocaine and household/industrial agents led to more severe outcomes. The overall ICU mortality rate was 5.1% (hospital mortality 6%) with a median ICU length of stay of 3 days. Early recognition of intoxication was associated with higher hospital survival rates, whereas lower pH and higher lactate levels were associated with increased hospital mortality.

**Conclusions:** Prompt identification of acute intoxications significantly impacts ICU and hospital outcomes. The findings suggest that early intervention, along with standardized treatment protocols, is crucial to improving patient prognosis and reducing mortality.

## 1. Background

Acute intoxications represent a complex yet under-researched area of critical care, likely due to the fact that they present a significantly lower mortality compared to general ICU cases [1]. However, a subset of these cases presents with life-threatening complications, such as organ failure, that demand rapid and invasive interventions, particularly in emergency and intensive care unit (ICU) settings.

The epidemiological impact of intoxication is also significant, yet frequently underestimated. For instance, poisoning is the leading cause of injury-related death in the United States [2, 3], surpassing fatalities from car accidents [4, 5]. Over the past 2 decades, poisoning-related deaths have risen sharply, with severe intoxications now representing 1%–3% of ICU admissions [6] and contributing to significant morbidity. These cases result in an average hospital stay of 3 days [7, 8] and a mortality rate between 1% and 5% [1, 9].

In Italy, specific epidemiological data on poisoning are limited. National death registers categorize fatal poisonings together with “external traumatic causes” of death, hindering precise assessment of intoxication-related fatality rates. However, recent data from the Italian National Institute of Statistics (ISTAT) show that, collectively, poisonings and trauma rank as the seventh leading cause of death [10].

Despite data underscoring the significant impact of acute intoxications, awareness of this issue remains limited. This is reflected in the relatively scant attention toxicology receives in medical literature [11], contributing to a lack of official guidelines—a gap that contrasts with the progress seen in other areas of evidence-based medicine.

In this context, our study aimed to provide a descriptive analysis of acute intoxication cases requiring ICU admission at our institution. Additionally, we sought to identify key factors that influence clinical outcomes.

## 2. Methods

### 2.1. Study Design

We conducted a retrospective cohort study at Fondazione IRCCS San Gerardo dei Tintori Hospital in Monza, Italy. The study included patients admitted to one of three ICUs (general ICU, cardiac ICU, and neuro ICU) for confirmed acute intoxication between January 2009 and May 2024.

Following local regulations, no ethics committee approval is required for retrospective epidemiological studies using healthcare administrative databases for research purposes with anonymized data. We followed STROBE reporting guidelines [12] for observational studies.

### 2.2. Data Collection

Data were gathered from electronic health records using specific keywords related to acute intoxication within the medical archives of the three ICUs. For each eligible patient, this entailed a comprehensive review of clinical history, focusing on:

• Demographic information: age, gender, and comorbidities
• Details of intoxication: substance(s) involved, circumstances of intoxication (voluntary or accidental)
• The severity of the condition at the time of ICU admission: initial laboratory results, clinical assessment
• Treatment: decontamination measures, use of antidotes, invasive interventions, supportive care
• Clinical outcomes: ICU and hospital length of stay and mortality

### 2.3. Inclusion and Exclusion Criteria

We included patients who met the following inclusion criteria: (1) The intoxication was caused by any xenobiotic, including pharmaceuticals, substances of abuse, chemical and natural toxins, either taken voluntarily or accidentally; (2) the toxic effect of a substance was the primary reason for ICU admission, regardless of whether the patient arrived from home or was already hospitalized for other reasons. Both adult and pediatric cases (age 1–17) were included in the sample.

Evidence of intoxication was based on anamnestic clues, toxicological assays, response to specific antidotes, or autopsy findings. In accordance with our institutional protocol, blood alcohol concentration was measured only in patients presenting in a comatose state of undetermined origin or in trauma patients. Standard toxicology screening performed by our laboratory includes assays for benzodiazepines and opioids. The identification of less common or specific substances (e.g., sodium nitrite) required targeted testing performed in specialized external laboratories, often in coordination with regional poison control centers. The reference Poison Center was located at ASST Grande Ospedale Metropolitano Niguarda in Milan, with additional toxicological analyses conducted at the Laboratory of Clinical Toxicology of ICS Maugeri in Pavia. The detected substances were classified according to their primary use or effects, such as “medication,” “illicit drug,” “household/industrial agents,” and “other.”

Specific attention was given to whether intoxication resulted from a single substance or multiple substances, to assess the association between the number of toxic agents and patient outcomes, as well as ICU resource utilization.

To ensure the independence of data points, only the first admission for acute intoxication for each patient was included in this study. Subsequent admissions were excluded from the analysis to avoid potential bias introduced by repeated measures. However, for completeness, a brief description of these repeated admissions is provided in the Results section.

### 2.4. Statistical Analysis

Categorical variables were reported as absolute and relative frequencies or proportions, while continuous variables were described using medians and interquartile ranges (25th–75th percentile). The population was stratified into two groups based on the number of substances involved: single- versus multiple-substance intoxications. Categorical variables were compared between groups using the chi-square or Fisher's exact test, and differences between continuous variables were investigated with Student's *T*-test or the Wilcoxon rank-sum test, as appropriate. To assess the association between variables and hospital mortality, univariable regression analyses were performed. The strength of association between covariates and outcomes was expressed as odds ratios with their corresponding 95% confidence intervals. Statistical significance was determined at a two-tailed alpha level of < 0.05. All analyses were performed using JMP 16 (SAS, Cary, NC).

## 3. Results

### 3.1. Patient Characteristics

Over the study period, 117 patients were admitted to the ICU for poisoning and were included in the study. Nine episodes were excluded because they involved individuals with multiple admissions, with six patients accounting for two to four admissions each. Only the first admission for each individual was included in the analysis.

Most patients (83, 70.9%) were admitted to the general ICU, with admissions peaking between 2015 and 2017; 22 patients (18.8%) were treated in the neuro ICU and 12 patients (10.3%) in the cardiac ICU.  Table 1 provides the demographic and clinical characteristics of the sample. There were no significant differences among the populations admitted to the three ICUs (see Table S1—Supporting Information). Ninety-two patients were admitted to the ICU directly after emergency department (ED) presentation. Of the remaining patients, 24 spent a median of 1 (IQR 1–2) days in the ED or in the general wards of our hospital before ICU admission. One patient was transferred from another public hospital.

The most frequent presentation involved voluntary self-poisoning in middle-aged women with mental disorders.

Across the 117 intoxications, 91 different substances, in various combinations, were detected. A total of 85 admissions (72.7%) were attributed to the misuse of medications, either alone or in combination with other toxins. The complete list of xenobiotics is provided in the Supporting Information (Table S2). A total of 52 cases (44.4%) involved a single substance, while 65 (55.6%) resulted from multiple substances. The distribution of different classes of substances is displayed in Table 2.

**Figure 1:**
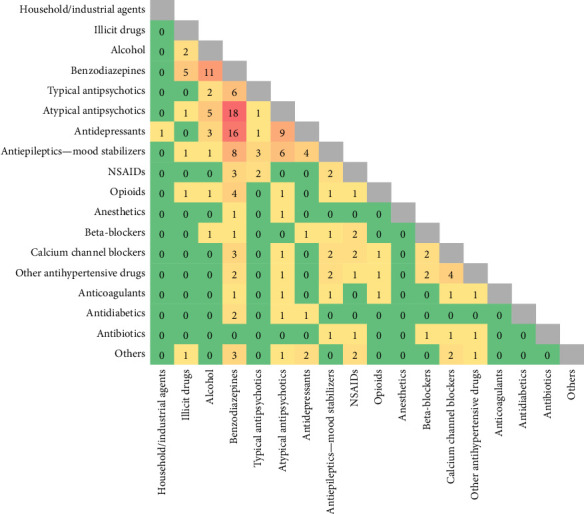
Combinations of drugs in multiple-substance intoxications. Values represent counts.

The most commonly involved classes of substances in acute intoxications were benzodiazepines, followed by atypical antipsychotics and antidepressants. These classes were also the most frequently combined in multiple-substance intoxications (see Figure 1).

Ethanol was frequently implicated, either alone or in combination with other substances. Other notable classes included antiepileptics/mood stabilizers and nonsteroidal anti-inflammatory drugs. Less commonly, opioids, household/industrial agents, and typical antipsychotics were involved.

### 3.2. Clinical Management

In 98 cases (83.8%), the initial clinical assessment accurately identified the intoxication (Table 1). Of the 19 cases where the initial assessment was inconclusive, 14 were attributable to the unavailability of toxicological test results.  Table 3 presents the treatments administered to the study population and the clinical outcomes. Decontamination procedures were performed in 81.2% of the admissions, mainly after consulting the Poison Center. Specific antidote therapy was administered to approximately 40% of patients, with flumazenil being the most frequently used treatment.

Table S3 (Supporting Information) presents further specifics regarding the intensive treatments administered, along with their median duration and the interval between hospitalization and initiation. The most required interventions were endotracheal intubation and vasopressors. Additional treatments were reserved for the most severe cases, particularly following specific intoxications. For instance, CRRT was primarily used in cases of metformin, lithium, and ethylene glycol poisoning. Three patients required extracorporeal membrane oxygenation (ECMO) support. One patient needed venoarterial ECMO for cardiogenic shock due to beta-blocker intoxication. Another patient received veno-venous ECMO due to cocaine intoxication (i.e., crack lung): Inhalation of cocaine triggered a marked inflammatory response, leading to the development of severe acute respiratory distress syndrome (ARDS) that proved refractory to standard treatments. The third case involved voluntary ingestion of sodium nitrite. The patient was found at home unconscious, cyanotic, and hypotensive and subsequently developed a refractory cardiac arrest. Venoarterial extracorporeal membrane oxygenation (VA-ECMO) was initiated but failed to improve the clinical condition. All supportive measures were withdrawn due to futility. The toxic agent was identified only through postmortem toxicological analysis. Only the first patient survived.

### 3.3. Outcomes and Predictors

Of the 117 cases of severe acute intoxication, 110 resulted in patient discharge and 7 in death (6 in the ICU and 1 in the surgery ward), corresponding to an ICU fatality rate of 5.1% and a hospital fatality rate of 6%. The median ICU stay was 3 days (IQR 1–6 days). After clinical stabilization in the ICU, 44 patients (37.6%) were transferred to the psychiatric ward. The median total hospitalization lasted 11 (5–18) days.

Table S4 (Supporting Information) details the characteristics of hospital nonsurvivors. Nonsurvivors were more frequently characterized by “first-time, single-substance” intoxications in elderly men not suffering from a mental disorder. Cocaine, metformin, and industrial agents were the substances most commonly involved in these fatal cases.

According to the univariable regression analysis (Table 4), early recognition of intoxication was significantly associated with reduced hospital mortality. Conversely, older age, lower pH, and higher lactate levels were associated with an increased risk of death.

### 3.4. Single- Versus Multiple-Substance Intoxication

The comparison of the study population, stratified by the number of substances involved (i.e., single- vs. multiple-substance intoxication), is provided in Tables 1 and 3. Patients exposed to a single substance had more medical comorbidities, whereas patients who ingested multiple substances were more likely to have mental disorders, with most having a history of prior intoxication, primarily related to medication abuse. In this group, all intoxications were voluntary. Alcohol was the most frequently involved substance in single-substance intoxications (8 cases, 6.8%), followed by metformin, which was implicated in 6 cases (5.1% of episodes, 2 of which were lethal).

Regarding severity, distinct trends emerged: Patients exposed to a single substance exhibited significantly higher lactate levels and a trend toward lower pH. Specific toxicological treatments also differed, with all decontamination procedures (except forced diuresis) being significantly more common among patients with multiple intoxications. Intensive interventions were generally balanced between the groups, although CRRT was significantly more frequent in single-substance intoxications.

These differences resulted in a trend toward longer hospital stays and higher ICU mortality among patients with single-substance intoxication, who also showed an increased hospital fatality rate.

### 3.5. Recurrent Intoxications

During the observation period, 6 patients presented at our institution multiple times (ranging from 2 to 4 visits) for acute intoxication, resulting in a total of 9 additional episodes.

Recurrent intoxications predominantly involved middle-aged women (median age 52 years, 83% females). All patients had a history of mental disorders. At the time of their first episode, 2 of them were not on psychoactive medications. Following their initial self-poisoning attempt, therapy was initiated; in both cases, subsequent intoxications involved the newly prescribed drugs.

All cases consisted of voluntary multiple-drug ingestion, with combinations of psychotropic medications being the most common. The time between recurrent episodes varied widely, ranging from a few months to as long as 10 years. Upon admission, the severity of recurrent intoxications was comparable to that observed in the first-episode sample. In all cases, intoxication was promptly recognized, and decontamination procedures were initiated immediately. All patients with recurrent episodes required intubation, with two needing vasopressors and one CRRT. The mean ICU and hospital stays were 3 and 14 days, respectively. All patients survived both the ICU and the hospital.

### 3.6. Pediatric Cases

Eight pediatric cases were included. Adolescents comprised the most common age group, although two infants were also involved. Seven cases (87.5%) resulted from voluntary intake, with ethanol and psychoactive drugs being the most commonly involved substances. One patient required endotracheal intubation and vasopressor support. All patients survived.

## 4. Discussion

In this retrospective cohort study, we analyzed the characteristics and outcomes of critically ill patients admitted to the ICU following acute intoxication. Our findings revealed that the majority of these patients were admitted due to intentional intoxications, often involving the ingestion of multiple substances, particularly psychotropic medications and ethanol.

The demographic characteristics of our study population revealed a predominance of middle-aged individuals, which may reflect the specific profile of severe intoxications requiring ICU admission. While some epidemiological studies report a younger population affected by acute poisoning [13, 14], others have shown slightly lower or comparable age medians [1, 9, 15]. These variations likely reflect differences in clinical settings, substances involved, and the severity threshold for hospital or ICU admission.

Despite the high demand for invasive mechanical ventilation and vasopressors—73% and 37%, respectively—the ICU and hospital mortality rates were relatively low. These rates compare favorably with the average fatality rate observed in our ICUs for any cause (approximately 5% vs. 15%–20%). These findings are consistent with the data reported in the literature and closely align with results from a recent multicenter study [5, 9, 16, 17].

One factor that may explain the relatively low number of fatal events in this study is that most intoxication cases involved combinations of ethanol and medications, such as psychotropic drugs, which typically have a high safety profile [14]. These medications are often prescribed to vulnerable populations, such as individuals with mental health conditions. Newer-generation drugs prioritize safety, resulting in fewer adverse events compared to older medications, even when taken in doses above the recommended range. This observation provides reassurance regarding the use of psychoactive drugs in patients at high risk of suicide, addressing a paradox in psychiatry: Medications intended to treat mental illness can, during episodes of decompensation, become potential means of self-harm. However, despite this concern, the fatality rate in this subgroup of patients remained notably low.

Another factor that may paradoxically reduce lethality in this sample is that most intoxication cases involved patients with mental disorders, mainly with voluntary ingestions as suicide attempts or demonstrative acts. In these cases, a self-preservation mechanism may emerge, as many patients self-report their substance abuse, enabling timely medical intervention [18, 19]. As highlighted in the literature, prompt identification and reporting of acute intoxication are crucial, particularly in cases with nonspecific clinical presentations or atypical substances [20, 21]. A recently published editorial proposed a toxidrome-based approach as a pragmatic diagnostic strategy for unknown poisonings, often allowing for rapid treatment despite limited available information [22]. Consistent with these findings, our study revealed that early recognition of intoxication was significantly associated with improved hospital survival outcomes.

However, the literature lacks a complete consensus on the factors that most significantly influence outcomes in intoxication cases. Various studies suggest that changes in vital signs, laboratory findings, or central nervous system (CNS) function may have the greatest impact, though results vary across case studies [17, 23]. Overall, the heterogeneity of intoxications limits the identification of prognostic factors, as evidenced by the low applicability of prognostic scores in clinical practice [24].

Interestingly, patients exposed to multiple substances showed better outcomes compared to those with single-substance intoxications. It may be explained by (1) the intrinsic low toxicity of the poison and (2) the prompt recognition of intoxication. Single-substance intoxications are often due to substances that produce long-lasting metabolic effects, sometimes with a delayed onset, while combinations of sedatives (the most common form of multiple intoxication) tend to have immediate effects, primarily limited to the suppression of CNS functions, which can sometimes be completely reversible after the toxin is eliminated. Furthermore, single-substance intoxications often coincide with an acute reduction in excretory functions, leading to the accumulation of the substance that develops over days. This poisoning mechanism limits the opportunity for early identification of the intoxication and effective decontamination measures, thereby reducing the ability to manage the clinical effects of the toxin.

These results contrast with the existing literature that identifies multiple-substance intoxications as the most lethal ones [25, 26]. The differences between these findings and ours may be attributed to the fact that in both reported studies, single-substance intoxications were primarily associated with ethanol, which is less lethal than substances such as cocaine, household/industrial agents, or metformin found in the single-substance intoxications in our sample.

The confirmed relatively low fatality rate of poisonings caused some authors to question whether the ICU was the most appropriate setting for treating these conditions, as in many cases, interventions were primarily limited to monitoring vital parameters [27]. Data from our sample generally align with this trend: Intensive procedures, apart from endotracheal intubation, were only required for a minority of patients, though purely observational hospitalizations were rare.

Recent literature has supported the conservative treatment of intoxications, emphasizing the importance of carefully selecting patients for specific toxicological interventions [17] and even suggesting noninvasive airway management in cases of poisoning [15]. However, these findings regarding airway management did not seem directly applicable to our sample. Specifically, poisonings involving psychoactive drugs—the most common in our study—may impair CNS functions in ways distinct from ethanol, the primary toxin studied in the NICO trial. This difference could necessitate more immediate airway control, which might have been delayed in the context of the French study setting. Furthermore, in our clinical practice, nasogastric tube insertion for decontamination was far more commonly used compared to the French trial, often requiring intubation to prevent the risk of aspiration.

Overall, the management of acute intoxications proved effective in most cases. However, to avoid underestimating their severity, survival data following ICU discharge should also be considered. A follow-up analysis was not included in the study design, but evidence from other centers suggested that the 5-year mortality rate among patients admitted to the ICU for poisoning was not only higher than that of the general population but also the highest among those admitted to the ICU for any cause [28].

In this context, it is important to note that 2 of the 7 fatal cases in the study were due to metformin toxicity, resulting from the accumulation of the drug taken at a constant dosage despite dehydration, vomiting, or gastroenteritis in elderly patients. These cases warrant particular attention, as such fatalities could have been prevented with proper medical prescriptions and better education for both patients and caregivers.

### 4.1. Limitations

This study has several limitations. First, its retrospective observational design, relying on case reporting, may have led to an underestimation of the true incidence of intoxications, as some cases could have been missed due to misidentification in medical records. Additionally, this design does not allow for establishing definitive causal relationships between recorded events and presumed contributing factors, limiting the reliability of the stratified analysis. The sample size, particularly the number of deaths, was insufficient for performing a multivariable analysis.

However, the extensive data collected for each patient provided a comprehensive examination of various aspects of intoxication management and enabled us to suggest potential relationships between factors influencing outcomes.

## 5. Conclusions

Severe acute intoxications remain a relatively under-researched area in medicine, despite their significant epidemiological impact. This study found that the most common poisoning scenarios involved the voluntary intake of psychotropic drug combinations, while the most lethal cases were associated with single-substance intoxications. Early recognition of poisoning was linked to reduced mortality. Further research is essential to improve prevention and management strategies, which could help lower mortality rates.

## Figures and Tables

**Table 1:** Demographic and clinical characteristics of the overall population and after stratification based on the number of substances.

	All (*N* = 117)	Single substance (*N* = 52)	Multiple substance (*N* = 65)	*p*
Sex (males)	54 (46.2%)	26 (50.0%)	28 (43.1%)	0.557
Age (years)	49 (36–59)	50 (34–71)	48 (38–56)	0.410
Medical comorbidities	68 (58.1%)	36 (69.2%)	32 (58.1%)	**0.029**
Mental/substance use disorders	87 (74.4%)	29 (55.8%)	58 (89.2%)	**< 0.0001**
Depression	50 (42.7%)	17 (32.7%)	33 (50.8%)	**0.050**
Anxiety	18 (15.4%)	4 (7.7%)	14 (21.5%)	**0.043**
Psychotic disorders	11 (9.4%)	7 (13.5%)	4 (6.2%)	0.213
Bipolar disorder	12 (10.3%)	3 (5.8%)	9 (13.9%)	0.222
Personality disorder	28 (23.9%)	6 (11.5%)	22 (33.9%)	**0.005**
Eating disorder	3 (2.6%)	1 (1.9%)	2 (3.1%)	0.999
Alcohol use disorder	16 (13.7%)	6 (11.5%)	10 (16.2%)	0.547
Illicit drug use disorder	16 (13.7%)	4 (7.7%)	12 (18.5%)	0.110
Previous episodes	44 (37.6%)	12 (23.1%)	32 (49.2%)	**0.004**
Accidental intake	19 (16.2%)	18 (34.6%)	0 (0%)	**< 0.0001**
Identification of poisoning	98 (83.76%)	38 (73.08%)	60 (92.31%)	**0.005**
Clinical parameters at admission				
FiO_2_	40 (21–70)	33 (21–95)	40 (21–60)	0.977
Arterial pO_2_	93 (63–240)	84 (62–217)	100 (67–263)	0.426
Arterial pCO_2_	42 (34–48)	41 (30–46)	44 (37–50)	0.083
Arterial pH	7.33 (7.25–7.38)	7.29 (7.21–7.38)	7.33 (7.28–7.38)	0.170
Lactates (mmol/L)	2.1 (1.5–4.5)	3.0 (1.8–7.4)	2.0 (1.3–3.1)	**0.006**

*Note:* Data are presented as frequencies and proportions (% of total or % of subgroup) or as median and interquartile range (25°–75°). FiO_2_ = inspiratory oxygen fraction; pO_2_ = partial pressure of oxygen; pCO_2_ = partial pressure of carbon dioxide. *p* values in bold indicate statistical significance (*p* < 0.05).

**Table 2:** Frequency and proportion of substance classes involved in acute intoxications.

	*N*	% of total
Benzodiazepines	49	41.9
Atypical antipsychotics	24	20.5
Antidepressants	24	20.5
Alcohol	23	19.7
Illicit drugs	15	12.8
Antiepileptics/mood stabilizers	13	11.1
Nonsteroidal anti-inflammatory drugs	9	7.7
Antidiabetics	9	7.7
Opioids	7	6.0
Household/industrial agents	6	5.1
Typical antipsychotics	6	5.1
Calcium channel blockers	6	5.1
Beta-blockers	4	3.4
Other antihypertensives	4	3.4
Anesthetics	2	1.7
Anticoagulants	1	0.9
Antibiotics	1	0.9

**Table 3:** Treatments and outcomes of intoxication in the overall population, stratified by the number of substances involved (single-substance vs. multiple-substance intoxications).

	All (*N* = 117)	Single-substance (*N* = 52)	Multiple-substance (*N* = 65)	*p*
Toxicological treatment				
Poison center contact	84 (71.8%)	28 (53.9%)	56 (86.2%)	**< 0.0001**
Decontamination	95 (81.2%)	38 (73.1%)	57 (87.7%)	**0.044**
Active charcoal	52 (44.4%)	15 (28.9%)	37 (56.9%)	**0.002**
Catharsis	43 (36.8%)	10 (19.2%)	33 (50.8%)	**0.0004**
Gastric lavage/EGD	66 (56.4%)	23 (44.2%)	43 (66.2%)	**0.018**
Forced diuresis	22 (18.8%)	8 (15.4%)	14 (21.5%)	0.397
Antidote	49 (41.9%)	16 (30.8%)	33 (50.8%)	**0.029**
ICU treatment				
Need for MV	85 (72.7%)	39 (75.0%)	46 (70.8%)	0.610
Tracheostomy	2 (1.7%)	1 (1.9%)	1 (1.5%)	0.999
ECMO	3 (2.6%)	2 (3.9%)	1 (1.5%)	0.584
CRRT	15 (12.8%)	11 (21.2%)	4 (6.2%)	**0.024**
Vasopressors	43 (36.8%)	22 (42.3%)	21 (32.3%)	0.265
Outcome				
ICU length of stay (days)	3 (1–6)	4 (1–7)	2 (1–5)	0.365
Hospital length of stay (days)	11 (5–18)	12 (5–20)	11 (5–16)	0.862
ICU mortality	6 (5.1%)	5 (9.6%)	1 (1.5%)	0.087
Hospital mortality	7 (6.0%)	6 (11.5%)	1 (1.5%)	**0.043**

*Note:* Data are presented as frequencies and proportions (% of total or % of subgroup) or as median and interquartile range (25°–75°). *p* values in bold indicate statistical significance (*p* < 0.05).

Abbreviations: CRRT, continuous renal replacement therapy; ECMO, extracorporeal membrane oxygenation; EGD, esophagogastroduodenoscopy; ICU, intensive care unit; MV, mechanical ventilation.

**Table 4:** Variables associated with hospital mortality by univariable logistic analysis.

	Odds ratio (95% confidence interval)
Sex (M, Ref. F)	1.6 (0.34–7.49)
Age (per unit)	1.05 (1.00–1.11)
Medical comorbidities (Ref. No)	4.64 (0.54–39.89)
Mental/substance use disorders (Ref. No)	0.23 (0.05–1.10)
Identification of poisoning (Ref. No)	0.07 (0.01–0.40)
Number of substances (single, Ref. Poly)	8.34 (0.97–71.71)
Type of intake (accidental, Ref. Voluntary)	2.35 (0.42–13.17)
Decontamination (Ref. No)	0.28 (0.06–1.35)
Admission pH (per 0.01 decrease)	1.05 (1.01–1.09)
Admission lactate (per unit increase)	1.17 (1.05–1.30)
Gastric lavage/EGD (Ref. No)	0.29 (0.05–1.55)
Use of antidotes (Ref. No)	0.53 (0.10–2.89)

*Note:* Associations are expressed as odds ratios with corresponding 95% confidence intervals.

Abbreviation: EGD, esophagogastroduodenoscopy.

## Data Availability

The data that support the findings of this study are available on request from the corresponding author. The data are not publicly available due to privacy or ethical restrictions.

## Supporting Information

This manuscript includes four supporting tables.

Table S1: Characteristics of the population stratified by ICU of admission (general, cardiac, and neuro intensive care units).

Table S2: Frequency of involved substances in the overall population.

Table S3: Intensive care treatments, duration, and interval from admission to their start.

Table S4: Description of characteristics in hospital survivors and nonsurvivors.

These tables provide additional data supporting the main findings of the study.
