# Supplementary material for: Acute Intoxications Admitted to the Intensive Care Unit: A Retrospective Cohort Study
Source: J Toxicol. 2025 Aug 6;2025:8823675. doi: 10.1155/jt/8823675 (PMC12349998; doi:10.1155/jt/8823675)
Supplement: Supporting Information — Additional supporting information can be found online in the Supporting Information section. [file 8823675.f1.docx]

**SUPPLEMENTARY MATERIAL**

**Acute Intoxications admitted to the Intensive Care Unit -**

**A Retrospective Cohort Study**

Martina Lombardo MD, Roberta Garberi MD, Emanuele Rezoagli MD, Matteo Pozzi MD, Francesco Bartoli MD, Roberto Rona MD , Giuseppe Citerio MD, Giuseppe Foti MD, and Marco Giani MD

**Table S1**: - Table S1– Characteristics of the population stratified by ICU of admission (General, Cardiac, and Neuro Intensive Care Units).

|  | **General ICU (N=83)** | **Neuro ICU (N=22)** | **Cardiac ICU (N=12)** | **p** |
| --- | --- | --- | --- | --- |
| **Sex (Males)** | 40 (48.2%) | 9 (40.9%) | 5 (41.7%) | 0.786 |
| **Age (years)** | 49 (36-59) | 49 (38-59) | 55 (31-61) | 0.983 |
| **Medical comorbidities** | 50 (60.2%) | 12 (54.6%) | 6 (50%) | 0.745 |
| **Mental / Substance Use disorders** | 61 (73.5%) | 17 (77.3%) | 9 (75%) | 0.934 |
| **Accidental intake** | 13 (15.7%) | 4 (18.2%) | 1 (8.3%) | 0.877 |
| **Multiple substance intake** | 46 (55.4%) | 13 (59.1%) | 6 (50%) | 0.851 |
| **Clinical parameters at admission** |  |  |  |  |
| FiO_2_ | 40 (21-83) | 45 (21-65) | 21 (21-60) | 0.702 |
| Arterial pO_2_ | 97 (62-260) | 142 (70-264) | 82 (54-93) | 0.539 |
| Arterial pCO_2_ | 44 (38-50) | 37 (33-47) | 36 (33-40) | 0.134 |
| Arterial pH | 7.3 (7.24-7.36) | 7.37 (7.32-7.42) | 7.34 (7.13-7.41) | 0.059 |
| Lactates (mmol/L) | 2.1 (1.5-4.1) | 2.8 (1.4-3.1) | 4.3 (1.5-9.5) | 0.269 |
| **Need for IMV** | 61 (73.5%) | 15 (68.2%) | 9 (75%) | 0.870 |
| **Vasopressors** | 35 (42.2%) | 5 (22.7%) | 3 (25%) | 0.150 |
| **ICU length of stay (days)** | 3 (1-7) | 2 (1-4) | 4 (1-6) | 0.306 |
| **Hospital length of stay (days)** | 11 (4-18) | 11 (4-21) | 13 (8-18) | 0.936 |
| **ICU mortality** | 6 (7.2%) | 0 (0%) | 0 (0%) | 0.119 |
| **Hospital mortality** | 6 (7.2%) | 1 (4.6%) | 0 (0%) | 0.410 |

Data are presented as frequencies and proportions (% of subgroup) or as median and interquartile range (25°-75°). p<0.05 (two-tailed) is considered statistically significant. Abbreviations: IMV, Invasive Mechanical Ventilation; ICU, Intensive Care Unit.

**Table S2**: Frequency of involved substances in the overall population (117 patients).

| **Acetylsalicylic Acid** | 4 (3.4%) | **Duloxetine** | 3 (2.6%) | **Mirtazapine** | 2 (1.7%) |
| --- | --- | --- | --- | --- | --- |
| **Alprazolam** | 13 (11.1%) | **Enalapril** | 1 (0.9%) | **Morphine** | 2 (1.7%) |
| **Aluminum Hydroxide** | 1 (0.9%) | **Escitalopram** | 4 (3.4%) | **Nail Polish Remover** | 1 (0.9%) |
| **Amanitine** | 1 (0.9%) | **Ethanol** | 24 (20.5%) | **Nebivolol** | 1 (0.9%) |
| **Amitriptyline** | 3 (2.6%) | **Ether** | 1 (0.9%) | **Olanzapine** | 4 (3.4%) |
| **Amlodipine** | 4 (3.4%) | **Ethylene Glycol** | 3 (2.6%) | **Olmesartan** | 2 (1.7%) |
| **Aripiprazole** | 3 (2.6%) | **Ethylephrine** | 1 (0.9%) | **Oxycodone** | 1 (0.9%) |
| **Atenolol** | 1 (0.9%) | **Etoricoxib** | 1 (0.9%) | **Paliperidone** | 1 (0.9%) |
| **Baclofen** | 1 (0.9%) | **Ezetimibe** | 1 (0.9%) | **Paracetamol** | 4 (3.4%) |
| **Benzodiazepines** | 11 (9.4%) | **Flurazepam** | 13 (11.1%) | **Paroxetine** | 4 (3.4%) |
| **Bisoprolol** | 2 (1.7%) | **Fluvoxamine** | 2 (1.7%) | **Perindopril** | 1 (0.9%) |
| **Bromazepam** | 3 (2.6%) | **Haloperidol** | 3 (2.6%) | **PfnPE (Herbicide)** | 1 (0.9%) |
| **Bupropion** | 3 (2.6%) | **Heroin** | 2 (1.7%) | **Prednisone** | 1 (0.9%) |
| **Cannabinoids** | 3 (2.6%) | **Hydralazine** | 1 (0.9%) | **Pregabalin** | 4 (3.4%) |
| **Carbamazepine** | 1 (0.9%) | **Hydrochloric Acid** | 2 (1.7%) | **Promazine** | 5 (4.3%) |
| **Carbon monoxide** | 2 (1.7%) | **Ibuprofen** | 1 (0.9%) | **Propranolol** | 1 (0.9%) |
| **Celecoxib** | 1 (0.9%) | **Indomethacin** | 4 (3.4%) | **Quetiapine** | 20 (17.1%) |
| **Ciprofloxacin** | 1 (0.9%) | **Insulin** | 2 (1.7%) | **Risperidone** | 1 (0.9%) |
| **Citalopram** | 9 (7.7%) | **Ivabradine** | 1 (0.9%) | **Ropivacaine** | 1 (0.9%) |
| **Clomipramine** | 1 (0.9%) | **Ketoprofen** | 2 (1.7%) | **Sertraline** | 3 (2.6%) |
| **Clonazepam** | 5 (4.3%) | **Lamotrigine** | 1 (0.9%) | **Sodium hypochlorite** | 3 (2.6%) |
| **Clotiapine** | 3 (2.6%) | **Laxatives** | 1 (0.9%) | **Sodium nitite** | 1 (0.9%) |
| **Clozapine** | 1 (0.9%) | **Levomepromazine** | 1 (0.9%) | **Sulfuric Acid** | 1 (0.9%) |
| **Cocaine** | 11 (9.4%) | **Lithium** | 5 (4.3%) | **Tapentadol** | 1 (0.9%) |
| **Codeine** | 1 (0.9%) | **Lorazepam** | 15 (12.8%) | **Trazodone** | 1 (0.9%) |
| **Colchicine** | 2 (1.7%) | **Lormetazepam** | 5 (4.3%) | **Valproic Acid** | 7 (6%) |
| **Dabigatran** | 1 (0.9%) | **Magnesium Hydroxide** | 1 (0.9%) | **Vitamin D** | 1 (0.9%) |
| **Degreaser** | 1 (0.9%) | **MDMA** | 1 (0.9%) | **Zofenopril** | 1 (0.9%) |
| **Delorazepam** | 8 (6.8%) | **Metformin** | 8 (6.8%) | **Zolpidem** | 4 (3.4%) |
| **Diazepam** | 4 (3.4%) | **Methadone** | 2 (1.7%) |  |  |
| **Diltiazem** | 1 (0.9%) | **Metoprolol** | 1 (0.9%) |  |  |

Data are presented as count and proportion. Abbreviations: MDMA=3,4-methylenedioxymethamphetamine.

**Table S3**: Intensive care treatments, duration and interval from admission to their start.

|  | **N**  **(% of the total)** | **Start time from ICU**  **Admission (days)** | **Duration(days)** | **Maximum dosage**  **(mcg/Kg/min)** |
| --- | --- | --- | --- | --- |
| **Need for MV** | 85 (72.7%) | 0 (0-0) | 2 (1-4) |  |
| **Tracheostomy** | 2 (1.7%) | 26 (21-30) |  |  |
| **ECMO** | 3 (2.6%) | 0 (0-0) | 5 (0-5) |  |
| **CRRT** | 15 (12.8%) | 0 (0-0) |  |  |
| **Vasopressors** | 43 (36.8%) | 0 (0-1) | 2 (1-3) |  |
| Norepinephrine | 40 (34.2%) |  |  | 0.17 (0.13-0.29) |
| Adrenaline | 6 (5.1%) |  |  | 0.11 (0.07-0.22) |
| Dopamine | 22 (18.8%) |  |  | 10.28 (5-16.36) |
| Dobutamine | 3 (2.8%) |  |  | 6.33 (3.91-14.53) |

Data are presented as count and proportion or as median and interquartile range (25°-75°). Abbreviations: CRRT, Continuous Renal Replacement Therapy; ECMO, Extracorporeal Membrane Oxygenation; MV, mechanical ventilation.

**Table S4:** Description of Characteristics in Hospital Survivors and Non-Survivors.

|  | **Survivors (N=110)** | **Non-survivors (N=7)** |
| --- | --- | --- |
| **Sex (M)** | 50 (45.5%) | 4 (57.1%) |
| **Age (years)** | 49 (36-58) | 71 (45-76) |
| **Mental /Substance Use Disorders** | 84 (76.4%) | 3 (42.9%) |
| **Previous episodes** | 42 (38.2%) | 2 (28.6%) |
| **Intake**  Voluntary  Accidental | 94 (85.5%)  16 (14.5 %) | 5 (71.4%)  2 (28.6%) |
| **Number of substances**  Mono-intoxication  Poly-intoxication | 46 (41.8%)  64 (58.2%) | 6 (85.7%)  1 (14.3%) |
| **Medication-Induced Poisoning** | 83 (75.5%) | 2 (28.6%) |

Data are presented as count and proportion or as median and interquartile range (25°-75°).
